# A Pilot Study Comparing HPV-Positive and HPV-Negative Head and Neck Squamous Cell Carcinomas by Whole Exome Sequencing

**DOI:** 10.5402/2012/809370

**Published:** 2012-12-12

**Authors:** Anthony C. Nichols, Michelle Chan-Seng-Yue, John Yoo, Wei Xu, Sandeep Dhaliwal, John Basmaji, Christopher C. T. Szeto, Samuel Dowthwaite, Biljana Todorovic, Maud H. W. Starmans, Philippe Lambin, David A. Palma, Kevin Fung, Jason H. Franklin, Bret Wehrli, Keith Kwan, James Koropatnick, Joe S. Mymryk, Paul Boutros, John W. Barrett

**Affiliations:** ^1^Department of Otolaryngology-Head and Neck Surgery, Western University, Victoria Hospital, London Health Science Centre, Room B3-431A, 800 Commissioners Road East, London, ON, Canada N6A 5W9; ^2^London Regional Cancer Program, London, ON, Canada N6A 4L6; ^3^Lawson Health Research Institute, London, ON, Canada N6C 2R5; ^4^Department of Oncology, Western University, London, ON, Canada N6A 4L6; ^5^Department of Pathology, Western University, London, ON, Canada N6A 5C1; ^6^Informatics and Biocomputing Platform, Ontario Institute for Cancer Research, Toronto, ON, Canada M5G 0A3; ^7^Department of Biostatistics, University of Toronto, Toronto, ON, Canada M5T 3M7; ^8^Department of Microbiology and Immunology, Western University, London, ON, Canada N6A 5C1; ^9^Department of Radiation Oncology (MAASTRO), GROW-School for Oncology and Developmental Biology, Maastricht University Medical Center, P.O. Box 616, 6200 MD Maastricht, The Netherlands; ^10^Department of Medical Biophysics, University of Toronto, Toronto, ON, Canada M5G 2M9

## Abstract

*Background*. Next-generation sequencing of cancers has identified important therapeutic targets and biomarkers. The goal of this pilot study was to compare the genetic changes in a human papillomavirus- (HPV-)positive and an HPV-negative head and neck tumor. *Methods*. DNA was extracted from the blood and primary tumor of a patient with an HPV-positive tonsillar cancer and those of a patient with an HPV-negative oral tongue tumor. Exome enrichment was performed using the Agilent SureSelect All Exon Kit, followed by sequencing on the ABI SOLiD platform. *Results*. Exome sequencing revealed slightly more mutations in the HPV-negative tumor (73) in contrast to the HPV-positive tumor (58). Multiple mutations were noted in zinc finger genes (ZNF3, 10, 229, 470, 543, 616, 664, 638, 716, and 799) and mucin genes (MUC4, 6, 12, and 16). Mutations were noted in MUC12 in both tumors. *Conclusions*. HPV-positive HNSCC is distinct from HPV-negative disease in terms of evidence of viral infection, p16 status, and frequency of mutations. Next-generation sequencing has the potential to identify novel therapeutic targets and biomarkers in HNSCC.

## 1. Introduction

Tobacco use has steadily declined over the last four decades [1]. In parallel, there has been a decline in cancers of most sites in the upper aerodigestive tract [2]. The exception to this trend is cancers of the oropharynx, particularly those of the palatine and lingual tonsils, which are caused by oncogenic subtypes of the human papillomavirus (HPV) [3]. The rise in incidence of HPV-positive head and neck squamous cell carcinoma (HNSCC) has been dramatic, causing the rates of tonsillar cancer to increase by as much as threefold in some countries [3, 4]. HPV-positive patients experience markedly better survival, and their tumors are molecularly distinct from traditional head and neck cancers [5]. Overexpression of p16 and proteolysis of p53 are nearly universal in HPV-positive tumors, in contrast to frequent loss of p16 and point mutations in p53 that are found in HPV-negative cancers [5]. However, the specific mechanisms responsible for improved survival in HPV-positive patients have not been fully elucidated.

Next-generation sequencing has yielded important insights into the pathogenesis of other cancers by identifying biomarkers and therapeutic targets. High-throughput sequencing of HNSCC tumors has recently been reported, and NOTCH inactivation was the most significant finding [6, 7]. This pilot study aims to contrast the mutations seen in an HPV-positive and an HPV-negative tumor using whole exome sequencing and further our understanding about the mutations that define HNSCC.

## 2. Methods

### 2.1. Patient Selection and Tumor and Blood Sample Collection

Ethics approval was obtained from Western University Health Sciences Research Ethics Board. Informed consent was obtained from patients undergoing ablative surgery for head and neck cancer to have a portion of their tumor stored, a 10 mL blood sample taken, and their clinical parameters prospectively collected. Two patients were identified for this pilot study: a 49-year-old nonsmoking male with a T2N0 tonsillar cancer treated with transoral robotic surgery and neck dissection and an 81-year-old female with a history of heavy smoking with a T2N0 oral tongue cancer treated with partial glossectomy, neck dissection, and free flap reconstruction. Primary site tumor specimens were taken from the center of the resection specimen. Ten mL of venous blood were drawn intraoperatively into heparinized collection tubes.

### 2.2. p16 Immunohistochemistry

For each patient, a portion of the primary tumor was fixed in formalin and embedded in paraffin. The blocks were then sectioned (5 *μ*m thick). p16 immunohistochemistry was performed as previously described using a mouse monoclonal antibody against p16 (MTM Laboratories, Heidelberg, Germany) at 1 : 500 dilution [8]. Immunohistochemistry scoring was conducted by two study pathologists (BW and KK) blinded to HPV status and patient information. Scoring was as described by Begum et al. with strong and diffuse staining (>80 percent of tumor cells) regarded as a positive result, and negative if absent or focal [9].

### 2.3. DNA Extraction from Blood and Tumor Tissue

DNA was extracted from 10 mL of whole blood using the QIAmp Blood Maxi kit following instructions provided by the manufacturer (Qiagen, Valencia, CA, USA). DNA was extracted from approximately 25 mg of primary tumor using the AllPrep DNA/RNA/Protein kit (Qiagen).

### 2.4. *In Situ* Hybridization for Human Papillomavirus Testing

Slides were deparaffinized by immersion in xylene, rehydrated in alcohol, and rinsed in water. Slides were then treated with 20 *μ*g/mL proteinase K (Sigma, St. Louis, MO) for 30 minutes, followed by immersion in 0.3% H_2_O_2_ in methanol at room temperature for 20 minutes. Slides were then treated for 10 minutes with avidin solution followed by biotin solution. The DakoGenpoint (Dako, Carpinteria, CA, USA) biotinylated probe that identifies high-risk subtypes 16, 18, 31, 33, 35, 39, 45, 51, 52, 56, 58, 59, and 68 was added to the slides. The slides were then covered and heated to 92°C for 5 minutes and then incubated at 37°C for 18 hours. DNA-DNA hybrids were detected by successive incubation with 1 : 100 diluted primary horseradish peroxidase-conjugated streptavidin (streptavidin-HRP) for 15 min, with biotinyltyramide for 15 min, and with secondary streptavidin-HRP for 15 min. A cervical cancer was used as a positive control, and a tonsil specimen from a healthy child undergoing tonsillectomy for sleep apnea was used as a negative control. Punctuate hybridization signals localized to the tumor cell nuclei defined an HPV-positive tumor. Scoring was conducted by the two study pathologists (BW and KK).

### 2.5. PCR Confirmation of Patient HPV Status

Patient DNA was extracted from thin section tissue slices. Briefly, a single pathology slide from each patient was deparaffinized, and then the tumor tissue was scraped into a 1.5 mL eppendorf tube containing 50 *μ*L of Arcturus PicoPure extraction buffer, containing proteinase K (Applied Biosystems). The sample was digested at 65°C for 16 hours. The proteinase K was inactivated at 95°C for 10 minutes, and DNA was used directly in PCR. Primers were designed against unique regions of the E6-E7 loci of HPV type 16 and type 18 and synthesized by Sigma Genosys (Oakville, Canada, Table 1). Primers were also synthesized against GAPDH, a cellular gene used as a positive control for the PCR reactions. 0.2 *μ*L of DNA extracted from the tumor tissue was added to the appropriate reaction tubes. PCR products were amplified with DNA Phusion polymerase (Thermo Scientific, Nepean, Canada) in 20 *μ*L reactions following the manufacturer's instructions.

### 2.6. Exome Sequencing

Exome libraries were created at The Centre for Advanced Genomics (Toronto, Canada) according to the manufacturer's standard protocol for SOLiD library preparation (Applied Biosystems, Carlsbad, CA, USA). Three *μ*g of genomic DNA extracted from matched patient blood and tumor samples was sheared via sonication using the Covaris (S-Series) instrument. The ends of fragmented DNA were repaired and ligated to SOLiD P1 and A1 adapters provided in the Agilent Human All Exon 50 Mb Kit following the manufacturers protocol (Agilent, Santa Clara, CA, USA). The exomes were then captured using the Agilent Human All Exon 50 Mb kit, and the amplified library was purified with AMPure XP beads (Beckman Coulter Genomics, Danvers, MA). Sequencing was performed with the SOLiDToP Paired End Sequencing Kit (Applied Biosystems). The image data collected was analyzed using the ABI corona pipeline to generate DNA reads that were mapped to the reference human genome (UCSC's hg19) using BFAST [10].

### 2.7. Bioinformatics

Samples were processed as matched sets through the Genome Analysis ToolKit (GATK) v1.3-16 pipeline [11]. Samples were initially locally realigned using the IndelRealigner walker from the GATK package with known insertions and deletions found in dbSNP 135. This was followed by base quality recalibration from GATK. Default parameters were used for both steps except for SOLiD specific parameters in the recalibration step. Reads without any color space calls were marked as failing vendor quality and thus were removed from further downstream analysis. In addition, reads that had a reference base inserted into the reads due to inconsistent color space calls had those bases set to Ns with base qualities of zero. Finally variants were called and filtered using the GATK UnifiedGenotyper and VariantFiltration walkers again with default settings.

To be considered for further downstream analysis, a tumor variant had to have at least 8x coverage within the target regions 37,038,261 sites (71.86%) for the HPV-positive tumor and 39,150,091 sites (75.96%) for the HPV-negative tumor that met this criterion. In addition to coverage, the following requirements had to be identified by the VariantFiltration walker:

- variant quality equal to or greater than 30,
- variant confidence/quality by depth (QD) equal to or greater than 2.0,
- MQ0 < 4 and MQ0/(1.0 ∗ DP)) < 0.1, where MQ0 is the total mapping quality zero reads and DP is the unfiltered read depth.

A reference variant required a minimum read depth of 8x within the target region for further consideration (38,673,520 sites (75.03%) and 38,058,450 sites (73.84%) for HPV-positive and HPV-negative tumors, resp.). This presented 36,020,799 and 37,049,778 comparable sites in the HPV-positive and HPV-negative tumors. Using in-house custom Perl code, somatic variants within the targeted regions were identified. To be classified as a somatic variant the following conditions had to be met: (1) a tumor variant was identified by GATK that met the above filtration requirements and (2) the corresponding position in the normal sample had 8x coverage and did not have a GATK variant call. Somatic variants were annotated with refGene annotations (http://varianttools.sourceforge.net/Annotation/RefGene), and consequences were identified using ANNOVAR v2012-03-08 [12].

## 3. Results

### 3.1. p16 Immunohistochemistry and HPV Testing

**Figure 1:**
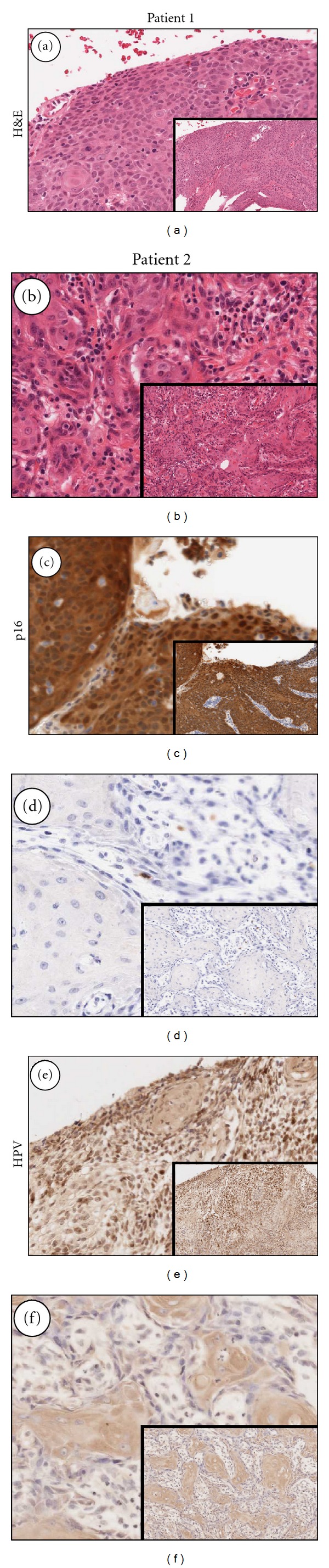
Tumors from two patients were sectioned. Slices were stained with ((a) and (b)) H&E, ((c) and (d)) p16, or ((e) and (f)) HPV *in situ* hybridization. Panels represent magnified images of the complete section (inset).

Genomic DNA was extracted from matching tumor and blood samples from two head and neck cancer patients: patient 1 was a 49-year-old nonsmoking and nondrinking male, and patient 2 was an 81-year-old female smoker. Patient demographics, treatment details, and histopathologic parameters are outlined in Table 2. Tumor sections from each patient were stained with hematoxylin and eosin (Figures 1(a) and 1(b)). Patient 1 stained diffusely positive for p16 (Figure 1(c)), while the tumor tissue from patient 2 was negative for p16 (Figure 1(d)). *In situ* hybridization testing with the broad-spectrum HPV probe demonstrated strong punctate staining within nuclei of the tumor of patient 1, consistent with high-risk HPV infection (Figure 1(e)). HPV-specific, punctate nuclear staining was absent in the tumor of patient 2 (Figure 1(f)).

**Figure 2:**
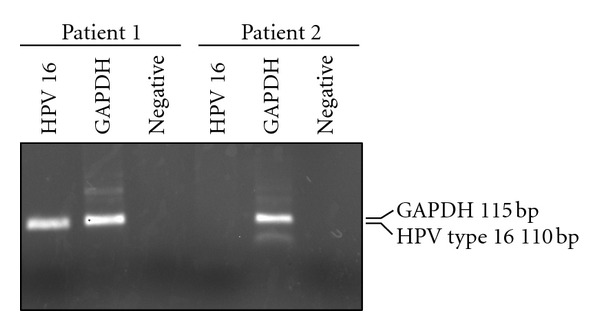
PCR confirmation of the presence of HPV type 16 DNA in patient 1 and the absence of HPV type 16 sequences in patient 2.

We employed primers designed specifically against unique portions of the E6-E7 region of HPV type 16 and type 18 to confirm the HPV status of the patients in this study. The GAPDH control was amplified from both patients; as expected, only patient 1 was HPV type 16 positive (Figure 2). Patient 2 was negative for HPV type 16, and both patients were HPV type 18 negative (data not shown).

### 3.2. Exome Capture and Raw Sequencing Results

The exomes from tumor tissue and matched blood samples from each patient were sequenced. For each tumor or blood sample, approximately 1.2 billion bases were sequenced, 86% of which were specific for exome sequences. The mean coverage of the exome targets was 28.1-fold, with 91.6% of the targets being sequenced at least once and 67.4% sequenced at least ten times. The exome capture and sequencing results were within the normal range of performance specified by the manufacturer and are comparable with published results [13].

### 3.3. Bioinformatic Interpretation of Sequencing Results

We compared the sequencing results of each patient's tumor to their matched blood samples in order to eliminate background germline variations and to focus on somatic alterations unique to the tumor genome. Although the exome capture is designed to target coding regions, some intergenic and intron regions adjacent exons are captured in the process. A complete listing of the identified variants in coding and noncoding regions for the HPV-positive and HPV-negative tumors is reported in Tables S1 and S2, respectively (see Supplementary Material available online at doi: 10.5402/2012/809370). Only the variants that occurred within exons are listed in Tables 3 and 4. Fifty-eight somatic mutations were noted in the HPV-positive tumor, 32 of which were nonsynonymous mutations within exons. Seventy-three mutations were observed in the HPV-negative tumor, including 36 coding mutations. Forty-nine of the mutated genes identified in this study were also shown to harbor mutations in large-scale sequencing studies (Tables S1 and S2) [6, 7].

No mutations were noted in TP53, CDKN2A (p16), or the NOTCH receptors in either tumor. However, multiple mutations were noted in zinc finger genes (ZNF3, 10, 229, 470, 543, 616, 664, 638, 716, and 799) and mucin genes (MUC4, 6, 12, and 16). Mutations were noted in MUC12 in both tumors.

**Figure 3:**
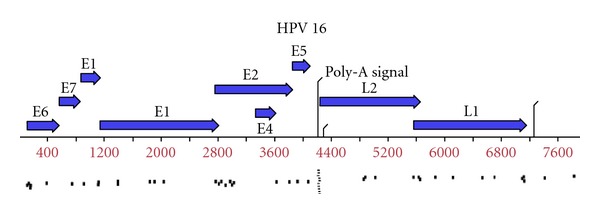
Detection of HPV 16 sequences with the exome captures of the four patient samples. Short read sequences generated from the exome sequencing data denoted by the small bars were found exclusively in the DNA from the HPV-positive tumor but not in the matched blood from the same patient or the HPV-negative patient's tumor or blood with the exception of the nonspecific poly-A signal.

Patient characteristics, PCR analysis, *in situ* hybridization testing, and immunohistochemistry all indicated that patient 1 was HPV type 16 positive (HPV type 18 negative by PCR) and that patient 2 was HPV negative (both type 16 and type 18 negative). When we used our four compiled exome sequences (blood and tumor from both patients) as queries against the HPV type 16 genomic sequence (RefSeq NC_001526.2), the tumor sequence from patient 1 matched numerous regions (39 hits) of the HPV 16 genome (Figure 3). Matches were identified to all the HPV type 16 genes (except E4) suggesting that the HPV type 16 genome had integrated into the tumor genome of patient 1. The tumor sample from patient 2 and the blood samples from both patients did not align to any specific HPV sequences.

## 4. Discussion

HPV-positive head and neck squamous cell carcinoma (HNSCC) has been described as molecularly distinct from traditional head and neck cancer [5]. The human papillomavirus (HPV) oncoproteins E6 and E7 promote carcinogenesis by degrading the tumor suppressors p53 and retinoblastoma protein (Rb), respectively. In contrast, p53 is not degraded in HPV-negative HNSCC but is frequently mutated, and p16 is often lost through homozygous deletion, methylation, or, less frequently, point mutation [5, 14]. This might lead one to believe that carcinogens like tobacco and alcohol would promote HNSCC comprised of a large number of mutations in many different pathways. In contrast HPV-positive cancers, modulated by the activities of viral oncoproteins, might not accumulate a large number of cellular mutations. In our study, we provided quadruple confirmation of tumor HPV status with p16 immunohistochemistry, HPV *in situ* hybridization, HPV detection by PCR, and detection of the HPV 16 genome sequences within patient 1's sequenced exome. We observed more mutations in the HPV-negative tumor when compared to the HPV-positive tumor, although the absolute difference was not dramatic (73 versus 58, resp.). Two large-scale exome sequencing efforts characterizing HNSCC have been reported recently [6, 7]. The study led by Stransky et al. reported twice as many mutations in the HPV-negative samples (4.83 mutations/Mb versus 2.28 mutations/Mb) [7]. The second group examined a set of 32 patients, four of which were HPV positive and reported on a subset of mutations that were identified by exome sequencing and confirmed by PCR. In this subset of genes, there were four times as many mutations in the HPV-negative tumors (20.6 ± 16.7 versus 4.8 ± 3 mutations in the HPV-positive tumors) [6]. Given the broad range of mutations seen in the HPV-negative cancers, our finding of slightly more mutations in the HPV-negative tumor is consistent with their results. As expected we did not identify TP53 or p16 mutations in the HPV-positive tumor; however these two genes appeared as wild type in the HPV-negative tumor as well. The lack of a p16 mutation in the setting of low expression levels as evidenced by immunohistochemistry may reflect that it has been inactivated by promoter methylation, the second most common cause of p16 loss [14].

Only a single genetic mutation (Muc12) was shared by both HPV-positive and HPV-negative tumor samples. The cell surface associated Muc12 was the only mucin identified in the HPV-negative tumor. In contrast, the HPV-positive tumor had five mutations in four different mucin genes, including the secreted Muc6, and the transmembrane bound Muc4, Muc12 and Muc16. Stransky et al. reported mutations in all the above mucins except for Muc12 [7]. Mucins are known to be involved in the development of epithelial cancer where they are often overexpressed, disrupting the epithelial cell polarity and promoting the epithelial to mesenchymal transition (EMT) phenotype [15]. Multiple damaging mutations within the mucins of HPV-positive tumor may suggest another cellular difference between these two distinct tumor types.

We also found multiple mutations in the zinc finger (ZNF) family genes in both tumor types. The ZNF family represents a large group of molecules which are involved in various aspects of transcriptional regulation [16]. There were almost twice as many ZNF mutated genes in the HPV-positive sample. Although there were a total of 11 ZNF mutations between the two tumor types, there were no shared ZNF members mutated in both cancers. Stransky et al. reported 50% of the same ZNF mutations that we found in both tumor samples suggesting that genetic changes to this family of transcription regulators may be important in the development of HNSCC [7]. Not enough is known about the role of mucins and ZNF proteins in HNSCC. These molecules may warrant further study.

We confirmed that the sequence from the human pathogen HPV type 16 was identified within exome sequence of a HNSCC tumor. In order for HPV to be oncogenic, the viral E2 protein, which represses the expression of E6 and E7, must be lost [17]. This only occurs during integration when the episomal HPV DNA breaks within the E2 gene. PCR detection of E6 and E7 can detect both episomal and integrated forms and thus cannot distinguish between a superficial HPV infection and integrated viral DNA causing the cancer [17]. An additional benefit of whole exome sequencing is the detection of integrated HPV DNA. In Stransky's study, exome sequencing appeared even more sensitive than PCR for detection of HPV, as it identified the presence of HPV 16 in 14 of 73 cases versus 11 for PCR [7]. Perhaps more interesting is the concept of screening human disease genomes against pathogen datasets. In fact, it was this exact strategy that led to the discovery of Merkel cell polyomavirus in 2008 [18]. It may be that a subset of other cancers have a yet undiscovered viral etiology.

This study represents a pilot effort to gain experience with this exciting new technology, which was instructive as our group moves forward with large-scale projects. In addition to the small sample size, the quality of data generated limited by the ABI SOLiD platform with an average 30-fold coverage with 50 base pair paired-end reads yielded only 10-fold coverage over approximately two-thirds of the coding sequence. Thus, approximately a third of the exome was not adequately evaluated and important mutations could have been missed. We have recently completed characterizing a panel of head and neck cancer cell lines with 100-fold coverage with 100 base pair paired-end reads, and the results were vastly superior [19]. An average of 99% of the targeted exome had at least 10 reads and 90% had fiftyfold coverage. Perhaps more importantly, an expert bioinformatics team is critical to properly analyze the data. Although there are standard steps involved with aligning the sequencing data to the reference genome, false positive results can be frequent without adequate quality control measures. A carefully validated pipeline is necessary to filter spurious results in order to generate valid data.

It should be noted that tremendous insights can be gained by exome sequencing; however, whole genome sequencing offers the advantage to identify other genetic changes that can lead to tumorigenesis including copy number variation and translocations, in addition to point mutations, insertions, and deletions. Alterations in noncoding regions that may be important, such as promoters and miRNAs, would also be identified. The study by Stransky et al. reported whole genome sequencing in two patients and revealed markedly more translocations in the HPV-negative versus the HPV-positive tumors [7]. Ideally, future large-scale initiatives will be carried out using this more extensive but also more expensive technique to identify additional important genetic changes underlying HNSCC.

## 5. Conclusions

Whole exome sequencing of head and neck cancers can provide important insights into the molecular biology of the disease. HPV-positive and negative head and neck cancers are molecularly distinct, and HPV-negative cancers tend to harbor more mutations. Multiple, integrated HPV 16 sequences were identified in the exome targets from the HPV positive patient. These matches were restricted to the HPV-positive patient's tumor profile providing evidence of the utility of screening exome sequences against pathogen databases.

## Supplementary Material

Supplementary Tables S1 and S2.
The supplementary tables provide a complete listing of the mutations identified by our bioinformatics pipeline in the HPV-positive (Table S1) and HPV-negative (Table S2) tumor samples but not in the matched blood samples. This listing includes mutations found in both coding and non-coding regions.

## Figures and Tables

**Table 1:** Primers for HPV testing.

Name	Sequence 5′ to 3′
GAPDH F	GCTCATTTGCAGGGGGGAGCC
GAPDH R	CTGATGATCTTGAGGCTGTTG
HPV 16 F	TTGCAGATCATCAAGAACACGTAGA
HPV 16 R	GTAGAGATCAGTTGTCTCTGGTTGC
HPV 18 F	CAACCGAGCACGACAGGAACG
HPV 18 R	TAGAAGGTCAACCGGAATTTTCAT

F: forward, R: reverse.

**Table 2:** Patient demographics.

	Patient 1	Patient 2
	HPV positive	HPV negative
Age	49	81
Gender	Male	Female
Primary site	Tonsil	Oral tongue
Stage	T2N0	T2N0
Smoking	Nonsmoker	50 pack years
Alcohol	Nondrinker	Rare
Differentiation	Moderate	Moderate to poorly
Adverse features	Perineural invasion	Perineural invasion
p16	Positive	Negative
HPV	Positive	Negative
Treatment	TORS + ND	Transoral resection, ND, RFFF

HPV: human papillomavirus, TORS: transoral robotic surgery, ND: neck dissection, and RFFF: radial forearm free flap.

**Table 3:** Coding mutations in the HPV-positive tumor.

Chr	Position	Reference allele	Tumor allele	Zygosity	dbSNP ID	Region	Type	Gene	Transcript name	Exon	CDS position	Protein change	Stansky*	Agrawal**
19	15789140	A	G	Homo	rs609290	Exonic	Nonsynonymous	CYP4F12	NM_023944	3	c.A268G	p.I90V	Yes	
10	88946876	G	A	Hetero		Exonic	Nonsynonymous	FAM35A	NM_019054	8	c.G2227A	p.D743N		
3	51930850	T	C	Homo	rs57859638	Exonic	Nonsynonymous	IQCF1	NM_152397	3	c.A169G	p.K57E		
7	20180717	C	G	Homo	rs3735615	Exonic	Nonsynonymous	MACC1	NM_182762	7	c.G2411C	p.R804T	Yes	
14	64882380	A	G	Hetero	rs1950902	Exonic	Nonsynonymous	MTHFD1	NM_005956	6	c.A401G	p.K134R		
7	100634194	C	G	Hetero		Exonic	Nonsynonymous	MUC12	NM_001164462	2	c.C350G	p.A117G		
19	9006667	A	G	Homo	rs75101943	Exonic	Nonsynonymous	MUC16	NM_024690	44	c.T39581C	p.I13194T	Yes	
19	9074122	T	G	Homo		Exonic	Nonsynonymous	MUC16	NM_024690	3	c.A13324C	p.T4442P	Yes	
3	195517753	C	T	Hetero		Exonic	Nonsynonymous	MUC4	NM_018406	2	c.G698A	p.G233E	Yes	
11	1017381	G	C	Hetero	rs34912894	Exonic	Nonsynonymous	MUC6	NM_005961	61	c.C5420G	p.T1807S	Yes	
19	50865535	A	G	Homo	rs676314	Exonic	Nonsynonymous	NAPSA	NM_004851	2	c.T119C	p.I40T		
4	170345835	G	C	Homo		Exonic	Nonsynonymous	NEK1	NM_001199397	31	c.C3091G	p.Q1031E		
17	3195485	C	T	Hetero		Exonic	Nonsynonymous	OR3A1	NM_002550	1	c.G392A	p.R131Q		
3	98073075	A	G	Hetero		Exonic	Nonsynonymous	OR5K4	NM_001005517	1	c.A378G	p.I126M	Yes	
5	140590766	A	G	Homo	rs2910006	Exonic	Nonsynonymous	PCDHB12	NM_018932	1	c.A2287G	p.K763E		
12	27787878	G	A	Hetero		Exonic	Nonsynonymous	PPFIBP1	NM_001198916	4	c.G100A	p.D34N		
15	43827261	G	C	Hetero		Exonic	Nonsynonymous	PPIP5K1	NM_001190214	30	c.C3832G	p.H1278D		
1	12853378	T	G	Hetero	rs112330886	Exonic	Nonsynonymous	PRAMEF1	NM_023013	2	c.T2G	p.M1R	Yes	
X	135956575	G	A	Hetero	rs78646793	Exonic	Nonsynonymous	RBMX	NM_002139	9	c.C902T	p.P301L		
6	33272855	G	C	Homo	rs2071888	Exonic	Nonsynonymous	TAPBP	NM_172208	4	c.C779G	p.T260R		
12	11183046	C	T	Hetero		Exonic	Nonsynonymous	TAS2R31	NM_176885	1	c.G889A	p.V297M		
12	11213969	A	G	Hetero	rs2599402	Exonic	Nonsynonymous	TAS2R46	NM_176887	1	c.T925C	p.S309P	Yes	
3	100084425	T	G	Homo		Exonic	Nonsynonymous	TOMM70A	NM_014820	12	c.A1810C	p.K604Q	Yes	
9	135277007	C	T	Homo	rs3739916	Exonic	Nonsynonymous	TTF1	NM_007344	2	c.G1202A	p.R401Q		Yes
19	38378659	A	T	Homo		Exonic	Nonsynonymous	WDR87	NM_031951	6	c.T5535A	p.N1845K		
9	74975645	C	G	Hetero		Exonic	Nonsynonymous	ZFAND5	NM_001102421	2	c.G50C	p.G17A		
12	133732818	C	T	Hetero		Exonic	Nonsynonymous	ZNF10	NM_015394	5	c.C986T	p.S329F		
19	44933706	C	T	Hetero	rs57014690	Exonic	Nonsynonymous	ZNF229	NM_014518	6	c.G1250A	p.S417N	Yes	
19	57089050	C	T	Hetero	rs4801177	Exonic	Nonsynonymous	ZNF470	NM_001001668	6	c.C1253T	p.T418I	Yes	
19	52618497	A	T	Hetero		Exonic	Nonsynonymous	ZNF616	NM_178523	4	c.T1920A	p.N640K	Yes	
2	71654175	G	A	Homo	rs1804020	Exonic	Nonsynonymous	ZNF638	NM_001252612	24	c.G5176A	p.V1726M		
7	57528923	G	C	Hetero		Exonic	Nonsynonymous	ZNF716	NM_001159279	4	c.G756C	p.W252C		

Chr: chromosome, *mutations present in Stransky et al., Science 2011 [7], **mutations present in Agrawal et al., Science 2011 [6], Homo: homozygous, Hetero: heterozygous.

**Table 4:** Coding mutations in the HPV-negative tumor.

Chr	Position	Reference allele	Tumor allele	Zygosity	dbSNP ID	Region	Type	Gene	Transcript name	Exon	CDS position	Protein change	Stansky*	Agrawal**
2	73679256	C	G	Homo		Exonic	Nonsynonymous	ALMS1	NM_015120	8	c.C5599G	p.L1867V	Yes	
10	37451586	G	A	Hetero	rs12766884	Exonic	Nonsynonymous	ANKRD30A	NM_052997	16	c.G1742A	p.G581E	Yes	
9	33385733	C	T	Hetero		Exonic	Nonsynonymous	AQP7	NM_001170	7	c.G657A	p.M219I		
17	42271663	G	C	Homo		Exonic	Nonsynonymous	ATXN7L3	NM_001098833	12	c.C1012G	p.P338A		
6	136599913	A	T	Hetero		Exonic	Nonsynonymous	BCLAF1	NM_001077441	4	c.T106A	p.S36T		
1	26646726	A	G	Hetero	rs1071849	Exonic	Nonsynonymous	CD52	NM_001803	2	c.A119G	p.N40S		
1	26646730	A	G	Homo	rs17645	Exonic	Nonsynonymous	CD52	NM_001803	2	c.A123G	p.I41M		
19	42213948	C	G	Hetero		Exonic	Nonsynonymous	CEACAM5	NM_004363	2	c.C414G	p.F138L		
12	31242362	A	G	Hetero	rs3950588	Exonic	Nonsynonymous	DDX11	NM_030653	8	c.A818G	p.K273R	Yes	
15	83657820	G	C	Hetero		Exonic	Stoploss	FAM103A1	NM_031452	3	c.G50C	p.X17S		
6	76023130	T	G	Homo		Exonic	Nonsynonymous	FILIP1	NM_015687	5	c.A2418C	p.Q806H,	Yes	
1	152191709	C	G	Hetero	rs6662450	Exonic	Nonsynonymous	HRNR	NM_001009931	3	c.G2396C	p.S799T	Yes	
9	21202136	A	G	Hetero	rs145794215	Exonic	Nonsynonymous	IFNA7	NM_021057	1	c.T29C	p.V10A		
7	100635103	A	C	Hetero		Exonic	Nonsynonymous	MUC12	NM_001164462	2	c.A1259C	p.E420A		
17	10404435	T	G	Hetero		Exonic	Nonsynonymous	MYH1	NM_005963	27	c.A3730C	p.K1244Q	Yes	Yes
18	47364086	C	T	Hetero		Exonic	Nonsynonymous	MYO5B	NM_001080467	37	c.G4939A	p.D1647N	Yes	
12	57106659	C	G	Hetero		Exonic	Nonsynonymous	NACA	NM_001113203	10	c.G2674C	p.E892Q	Yes	
1	21795333	C	G	Hetero		Exonic	Nonsynonymous	NBPF3	NM_032264	3	c.C286G	p.Q96E	Yes	Yes
10	115370274	T	C	Homo	rs10749138	Exonic	Nonsynonymous	NRAP	NM_006175	30	c.A3442G	p.I1148V	Yes	Yes
11	48346916	G	C	Homo	rs77069283	Exonic	Nonsynonymous	OR4C3	NM_001004702	1	c.G424C	p.V142L	Yes	
11	4608046	T	A	Hetero		Exonic	Nonsynonymous	OR52I2	NM_001005170	1	c.T4A	p.C2S		
11	56128081	A	G	Hetero	rs10896290	Exonic	Nonsynonymous	OR8J1	NM_001005205	1	c.A359G	p.Y120C	Yes	
11	56143823	G	A	Homo	rs77614949	Exonic	Nonsynonymous	OR8U1, OR8U8	NM_001005204	1	c.G724A	p.G242S		
X	82764042	G	C	Hetero	rs5921979	Exonic	Nonsynonymous	POU3F4	NM_000307	1	c.G710C	p.G237A	Yes	
19	43709656	C	G	Homo	rs11883278	Exonic	Nonsynonymous	PSG4	NM_213633	1	c.G33C	p.Q11H	Yes	
14	21511497	C	T	Homo	rs1243469	Exonic	Nonsynonymous	RNASE7	NM_032572	2	c.C346T	p.H116Y		
1	153004853	C	T	Homo	rs3795382	Exonic	Nonsynonymous	SPRR1B	NM_003125	2	c.C32T	p.T11I		
6	33410273	T	A	Homo		Splicing	NA	SYNGAP1	NM_006772	14	c.2336+2T>A			
12	11174327	C	T	Hetero	rs72475481	Exonic	Nonsynonymous	TAS2R19	NM_176888	1	c.G844A	p.G282R		
12	11183676	C	T	Hetero	rs73049072	Exonic	Nonsynonymous	TAS2R31	NM_176885	1	c.G259A	p.V87I		
7	35244086	A	T	Hetero		Exonic	Nonsynonymous	TBX20	NM_001077653	7	c.T999A	p.N333K		
21	10910347	A	G	Hetero	rs150482	Exonic	Nonsynonymous	TPTE	NM_199261	22	c.T1409C	p.L470P		
7	99669149	C	T	Hetero		Exonic	Nonsynonymous	ZNF3	NM_032924	6	c.G958A	p.A320T		
19	57839150	A	G	Homo	rs8100491	Exonic	Nonsynonymous	ZNF543	NM_213598	4	c.A320G	p.Q107R	Yes	
12	124497119	T	A	Hetero		Exonic	Nonsynonymous	ZNF664	NM_001204298	5	c.T428A	p.F143Y		
19	12501852	C	T	Hetero		Exonic	Nonsynonymous	ZNF799	NM_001080821	4	c.G1360A	p.G454R	Yes	

Chr: chromosome, *mutations present in Stransky et al., Science 2011 [7], **mutations present in Agrawal et al., Science 2011 [6], Homo: homozygous, Hetero: heterozygous.
